# Characterization of Four Novel Caspases from *Litopenaeus vannamei* (Lvcaspase2-5) and Their Role in WSSV Infection through dsRNA-Mediated Gene Silencing

**DOI:** 10.1371/journal.pone.0080418

**Published:** 2013-12-23

**Authors:** Pei-Hui Wang, Ding-Hui Wan, Yong-Gui Chen, Shao-Ping Weng, Xiao-Qiang Yu, Jian-Guo He

**Affiliations:** 1 MOE Key Laboratory of Aquatic Product Safety/State Key Laboratory of Biocontrol, School of Life Sciences, Sun Yat-Sen University, Guangzhou, People’s Republic of China; 2 School of Marine Sciences, Sun Yat-Sen University, Guangzhou, People’s Republic of China; 3 Division of Cell Biology and Biophysics, School of Biological Sciences, University of Missouri-Kansas City, Kansas City, Missouri, United States of America; Florida International University, United States of America

## Abstract

Apoptosis plays an important role in white spot syndrome virus (WSSV) pathogenesis, and caspases are central players in apoptosis. Here, we cloned four novel caspases (Lvcaspase2-5) from the Pacific white shrimp *Litopenaeus vannamei*, and investigated their potential roles in WSSV replication using dsRNA-mediated gene silencing. Lvcaspase2-5 have the typical domain structure of caspase family proteins, with the conserved consensus motifs p20 and p10. *Lvcaspase2* and *Lvcaspase5* were highly expressed in muscle, while *Lvcaspase3* was highly expressed in hemocytes and *Lvcaspase4* was mainly expressed in intestine. Lvcaspase2-5 could also be upregulated by WSSV infection, and they showed different patterns in various tissues. When overexpressed in *Drosophila* S2 cells, Lvcaspase2-5 showed different cellular localizations. Using dsRNA-medicated gene silencing, the expression of *Lvcaspase2*, *Lvcaspase3*, and *Lvcaspase5* were effectively knocked down. In *Lvcaspase2*-, *Lvcaspase3*- or *Lvcaspase5-*silenced *L. vannamei*, expression of WSSV *VP28* gene was significantly enhanced, suggesting protective roles for Lvcaspase2, Lvcaspase3 and Lvcaspase5 in the host defense against WSSV infection.

## Introduction

Apoptosis plays a protective role in eliminating harmful cells and in the host response to viral infections [1,2]. When virus-infected cells undergo apoptosis, the viruses already replicated in these cells are unable to diffuse and infect other cells [1,2]. Viruses have developed distinct strategies to escape or retard apoptosis triggered by various apoptotic pathways [1-3]. For instance, viruses can block apoptosis to prevent premature death of a host cell, thereby maximizing the viral progeny from a lytic infection or facilitating a persistent infection; in contrast, viruses can also actively promote apoptosis to spread viral progeny to neighboring cells [1-3]. Viruses may perform both pro- and anti-apoptotic functions to facilitate different stages of infection. 

Interference with apoptosis by inhibiting the proteolytic activity of cysteine aspartic acid proteases (caspases) prolongs the life of virus-infected cells, resulting in enhanced viral replication and viral persistence [4]. Caspases are a family of structurally related cysteine proteases, and they play a central role in apoptosis. Caspases contain three main domains, namely a prodomain, a large (p20, 20 kDa) catalytic subunit, and a small (p10, 10 kDa) catalytic subunit [5-7]. Based on their roles in apoptosis, the caspase family proteins are divided into two subgroups, initiator caspases and effector caspases [2,6]. The initiator caspases have a long prodomain (> 90 amino acids) containing specific protein-protein interaction motifs that are necessary for their activation, whereas the effector caspases usually have a short prodomain of only 20-30 residues [8]. Initiator caspases such as caspases 2, 8, 9, and 10 can be activated by autocatalysis in response to apoptotic signals [2,6,7]. Subsequently, the initiator caspases cleave and activate effector caspases such as caspases 3, 6, and 7 in a cascade [2]. Next, the effector caspases cleave many specific substrates and degrade numerous cellular proteins, leading to the disintegration of the entire cell contents into apoptotic bodies [2,7]. Some members of the caspase family of proteins, such as caspase-3, are key players in the virus-induced apoptosis [9,10]. The proper activation of caspase-3 is believed to be essential for efficient virus propagation during influenza infections [9].

White spot syndrome virus (WSSV) is one of the most common and destructive pathogens in shrimp aquaculture. Shrimp mortality can reach 100% 3-10 days after infection. WSSV infection induces apoptosis in bystander cells that are free of WSSV virions, while virion-containing cells are non-apoptotic [11-15]. Two WSSV anti-apoptosis proteins have been identified, AAP-1 (ORF390 or WSSV449) and WSSV222 [11,16,17]. WSSV449 bind to and is cleaved by *Penaeus monodon* (Pm) caspase, inhibiting Pm caspase activity *in vivo* and *in vitro* [18,19]. WSSV449 can also modulate NF-κB activity, which might be another way of inhibiting apoptosis during WSSV infection besides direct inhibition of Pm caspase activity [11,20]. WSSV222, an E3 ubiquitin ligase that acts through ubiquitin-mediated degradation, may function as an anti-apoptosis protein in WSSV-infected shrimp via ubiquitin-mediated degradation of a suppressor-like protein [11,17]. WSSV infection also actively modulates the expression of several shrimp apoptosis-related genes, including *PmCasp*, *PjCaspase*, *Pm-fortilin* and voltage-dependent anion channels (VDAC), to benefit viral multiplication [11,17,21-27]. Currently, two different effector caspase genes, *PmCasp* and *Pm caspase*, have been cloned from *P. monodon* [19,24]. PjCaspase from *P. japonicas*, the sole initiator caspase identified in shrimp, might also be upregulated by WSSV infection [22]. Many studies have indicated that WSSV-induced apoptosis represents an antiviral immune response in shrimp and that inhibition of apoptosis by the inhibitor zVAD-FMK or *PjCaspase* silencing would facilitate the multiplication of WSSV [11,22,28]. However, another group reported that silencing the *caspase3* gene of *L. vannamei* provided partial protection against WSSV infection [23]. To further investigate the contribution of shrimp caspases to host defense against WSSV infection, we cloned four novel caspases from *L. vannamei* and studied their expression profile, cellular localization and potential functions in WSSV infection. 

## Materials and Methods

### 2.1: Microorganisms and experimental shrimp

Gram-negative *Vibrio alginolyticus* and WSSV inocula were prepared as described previously [29-31]. Pacific white shrimp, *L. vannamei* (~8-10 g each for gene expression analysis; ~1-2 g each for dsRNA-mediated gene silencing), were purchased from a shrimp farm in Zhuhai, Guangdong Province, China. The shrimp were cultured in a recirculating water tank system filled with air-pumped seawater (2.5% salinity) at 24-26°C and were fed a commercial diet at 5% of their body weight twice daily. The shrimp were cultured for at least seven days to acclimate before beginning experiments.

### 2.2: Rapid amplification of cDNA ends

Total RNA (0.5 μg) was isolated from shrimp gills using an RNeasy Mini Kit (Qiagen, Germany) and reverse transcribed into cDNA using a SMARTer™ RACE cDNA Amplification Kit (Clontech, USA) for cloning the 5’ and 3’ cDNA ends of genes. Based on the expression sequence tag (EST) of *L. vannamei* in the NCBI database, the full-length cDNA sequences of *Lvcaspase2-5* were obtained using a RACE-PCR approach as described previously [29-32]. All conditions were as described except for the primer sequences (listed in Table 1). 

**Table 1:** PCR primers used in this study.

**Primer**		**Primer sequence (5’-3’)**
**cDNA cloning**		
5’ Lvcasp2-RACE1		TTGGAATCCCAGGTTAGTGAAG
5’ Lvcasp2-RACE2		ACCGTTGACAGTTTCCTCCATT
3’ Lvcasp2-RACE1		TCTTCAACCACCGCCACTT
3’ Lvcasp2-RACE2		TGGCTACCAGGCTTACAGATTC
5’ Lvcasp3-RACE1		CACCCCACCCTCTTCGTC
5’ Lvcasp3-RACE2		CACCATCGGGTATGTCAAGC
3’ Lvcasp3-RACE1		GGGCGGAACACCACTCAC
3’ Lvcasp3-RACE2		ATGCGGAAGACGAAGAGGG
5’ Lvcasp4-RACE1		TGGGGTCTTTTCCGCTCTT
5’ Lvcasp4-RACE2		CTTTCTCCAGTGCCCTTTGAT
3’ Lvcasp4-RACE1		ACCGACCTCATCCAACCATTC
3’ Lvcasp4-RACE2		ACCGAAAGAGGTTCTCGTCAAC
5’ Lvcasp5-RACE1		GGTCTTCAAAATCCTTGTCTCG
5’ Lvcasp5-RACE2		GAACTCCACATCAAGGGAAGAAT
3’ Lvcasp5-RACE1		TTCTTCCCTTGATGTGGAGTTC
3’ Lvcasp5-RACE2		TTATACAGGGAGGTCGAGGCG
**qPCR analysis**		
qPCR-Lvcasp2-F		ATGGCTCGTGGTTCATTCAG
qPCR-Lvcasp2-R		CATCAGGGTTGAGACAATACAGG
qPCR-Lvcasp3-F		AGTTAGTACAAACAGATTGGAGCG
qPCR-Lvcasp3-R		TTGTGGACAGACAGTATGAGGC
qPCR-Lvcasp4-F		CATGCTTGACATACCCGATG
qPCR-Lvcasp4-R		TGTCCGGCATTGTTGAGTAG
qPCR-Lvcasp5-F		GAAGGAGTGAAGCTAAACGAGAC
qPCR-Lvcasp5-R		CAGTAGACCAGCAGATAAGGAAGT
qPCR-LvEF-1α-F		GAAGTAGCCGCCCTGGTTG
qPCR-LvEF-1α-R		CGGTTAGCCTTGGGGTTGAG
dsRNA preparation*		
dsGFP-F		AGTGCTTCAGCCGCTACCC
dsGFP-R		GCGCTTCTCGTTGGGGTC
dsGFP(T7)-F		TAATACGACTCACTATAGGAGTGCTTCAGCCGCTACCC
dsGFP(T7)-R		TAATACGACTCACTATAGGGCGCTTCTCGTTGGGGTC
dsLvcasp2-F		ATCTTCAACCACCGCCACT
dsLvcasp2-R		AGTCAGCCGTGTTGGGAAT
dsLvcasp2(T7)-F		TAATACGACTCACTATAGGATCTTCAACCACCGCCACT
dsLvcasp2(T7)-R		TAATACGACTCACTATAGGAGTCAGCCGTGTTGGGAAT
dsLvcasp3-F		GACCTTGGCTTCATAGTGCG
dsLvcasp3-R		ACCATGAGCCGGTATTGGT
dsLvcasp3(T7)-F		TAATACGACTCACTATAGGGACCTTGGCTTCATAGTGCG
dsLvcasp3(T7)-R		TAATACGACTCACTATAGGACCATGAGCCGGTATTGGT
dsLvcasp5-F		GGTGAAGAGCGAGACTACCG
dsLvcasp5-R		TCCAATGCCTTGTGCGATA
dsLvcasp5(T7)-F		TAATACGACTCACTATAGGGGTGAAGAGCGAGACTACCG
dsLvcasp5(T7)-R		TAATACGACTCACTATAGGTCCAATGCCTTGTGCGATA
**Cellular localization**		
pA5.1Lvcasp2-F		CGGGGTACCATGGAGGAAACTGTCAACGGT
pA5.1Lvcasp2-R		GCTCTAGAATACTTTGGCGTAAAGTACACCTTT
pA5.1Lvcasp3-F		AAGGAAAAAAGCGGCCGCCGCCACCATGGACATCACAAT-CCAGGC
pA5.1Lvcasp3-R		GCTCTAGACCCTCTGCATCTCCTCACG
pA5.1Lvcasp4-F		CGGAATTCCGCCACCATGGTGATGAGGAAACAGCTCC
pA5.1Lvcasp4-R		GCTCTAGATTGACCCACGCCAGCCGC
pA5.1Lvcasp5-F		CGGGGTACCCGCCACCATGGTCCCGGACTTAGACTCTCT
pA5.1Lvcasp5-R		GCTCTAGAGTCCACTTCTTCGTCTTCTATATGTG

### 2.3: Bioinformatic analysis

Using the NCBI database, nucleotide blast searches were conducted to retrieve potential caspase-like ESTs. Multiple sequence alignments were performed using the ClustalX 2.0 program (http://www.ebi.ac.uk/tools/clustalw2). The simple modular architecture research tool (SMART; http://smart.embl-heidelberg.de) was used to analyze the domain structure of Lvcaspase2-5. Neighbor Joining (NJ) phylogenic trees were constructed using MEGA 4.0 software (http://www.megasoftware.net/index.html) based the on protein sequences of caspase family proteins in typical species. Bootstrap sampling was reiterated 1,000 times.

### 2.4: Sample preparation and real-time quantitative PCR

For tissue distribution studies, the hemocyte, eyestalk, gill, heart, hepatopancreas, stomach, intestine, nerve, muscle, pyloric cecum, and epithelium samples were collected from healthy *L. vannamei* to extract total RNA for first-strand cDNA preparation. For immune challenges, healthy *L. vannamei* were injected intramuscularly at the third abdominal segment with 2.4×10^6^ *V. alginolyticus* or 100 µl of WSSV inoculum (approximately 10^7^ copies/shrimp). PBS-injected shrimp were used as controls. At 0, 3, 6, 12, 24, 36, 48 and 72 hours post-injection (hpi), five shrimp from each group were randomly selected for the gill, hemocyte, hepatopancreas, intestine, and muscle sample collection. Shrimp total RNA isolation and preparation of cDNA templates for PCR were conducted as previously described [29-32]. Five-fold dilutions of cDNA templates were prepared, and 1 µl was used to detect the expression of *Lvcaspase2-5* in healthy and immune-challenged shrimp using the Master SYBR Green I system and a LightCycler (Roche) with the following program: 1 cycle of 95°C for 30 s and 40 cycles of 95°C for 5 s, 57°C for 20 s, and 78°C for 1 s. Three qPCR replicates were performed per sample, and three shrimp were analyzed for each sample. The expression of *L. vannamei* elongation factor 1α (*LvEF-1α*) was used as an internal control. Standard curves for *Lvcaspase*2-5 and *LvEF-1α* were generated by running triplicate reactions of a 10-fold dilution series (10 different cDNA concentrations). The primer amplification efficiencies for *Lvcaspase2*, *Lvcaspase3, Lvcaspase4, Lvcaspase5* and *LvEF-1α* were 1.943, 1.958, 2.019, 1.851 and 1.953, respectively. The relative standard curve method was used for calculation of the fold changes in gene expression [33-35]. 

### 2.5: Plasmid construction

The pAc5.1-N-GFP vector constructed in our previous study expressed sufficient green fluorescent protein (GFP) in *Drosophila* S2 cells [20,30,31,36]. For cellular localization of Lvcaspase2-5, PCR products containing the complete open reading frames (ORFs) of *Lvcaspase2-5* were inserted into pAc5.1-N-GFP using standard molecular cloning methods to construct the expression vectors pAc5.1-Lvcaspase2-5-GFP. 

### 2.6: Cell culture

*Drosophila* S2 cells were maintained at 28°C without CO_2_ in Schneider's *Drosophila* medium (SDM) supplemented with 10% fetal bovine serum (FBS) (Invitrogen, USA). When the culture density reached approximately 6-20 × 10^6^ viable cells ml^−1^, the *Drosophila* S2 cells were passaged onto a new plate at a density of approximately 5 × 10^5^ viable cells ml^−1^. 

### 2.7: Confocal microscopy analysis

*Drosophila* S2 cells were seeded onto poly-l-lysine-coated cover slips in 24-well plates. Approximately 24 hours later, cells were transfected with pAC5.1-Lvcaspase2-5-GFP. At 36 hours post-transfection, the cells on the cover slips were washed twice with PBS, fixed using Immunol Staining Fix Solution (Beyotime, China) and stained with Hoechst 33258 Solution (Beyotime, China). The cells on the cover slips were observed using a Leica laser scanning confocal microscope as previously described [30,31,36]. 

### 2.8: dsRNA preparation and dsRNA mediated gene silencing in vivo

Double-stranded RNA (dsRNA) sequences corresponding to *Lvcaspase2-5* and *GFP* (dsLvcaspase2, dsLvcaspase3, dsLvcaspase4, dsLvcaspase5, and dsGFP, respectively) were prepared using the T7 RiboMAX Express Kit (Promega, USA) as previously described [37]. In dsRNA-mediated gene silencing experiments, the experimental group (1-2 g per shrimp) was injected with dsLvcaspase2, dsLvcaspase3, dsLvcaspase4 or dsLvcaspase5 (1 µg/g shrimp) by intramuscular injection, while the control groups were injected with dsGFP or PBS. To evaluate silencing, gill samples from at least 3 shrimp in each treatment group were collected at 0, 24, 72, 120 and 144 hours post-dsRNA injection (hpi) for total RNA extraction. The first-strand cDNA prepared from the gill total RNA was used to detect gene silencing efficiency using qPCR as described in Section 2.5. 

### 2.9: WSSV infection experiments in dsRNA-injected *L*. *vannamei*

The gene silencing efficiency of *Lvcaspase2*, *Lvcaspase3*, and *Lvcaspase5* was significant compared with the control groups (> 80%) at all the examined time points. In the WSSV infection experiments, *L. vannamei* were infected intramuscularly with 100 µl WSSV inoculum (approximately 10^7^ copies/shrimp) at 48 hours post dsRNA injection (hpi), and gill samples were collected at 0, 3, 6, 12, 24, 36 and 48 hours post WSSV infection for detection of WSSV *VP28* expression. 

### 2.10: Statistical analysis

Student’s *t*-test was used to compare means between pairs of samples using Microsoft Excel. In all cases, differences were considered significant at p < 0.05 and highly significant at p < 0.01. The data are presented as the means ± standard error (standard error of the mean, SEM).

## Results

### 3.1: Cloning and sequence analysis of four novel caspases from *L*. *vannamei*

Based on the ESTs of *L. vannamei* in the NCBI database, the full-length cDNA sequences of four novel caspases were identified and named *Lvcaspase2*, *Lvcaspase3*, *Lvcaspase4* and *Lvcaspase5* after the reported *Lvcaspase1* (called *Penaeus vannamei cas-3* in the original report). The *Lvcaspase2* cDNA was 1,490 bp and contained a 924-bp ORF encoding a putative 307-amino acid protein, a 5’ untranslated region of 96 bp, and a 3’ untranslated region of 470 bp (Figure S1A). The *Lvcaspase3* cDNA was 2,083 bp and contained a 1,482-bp ORF encoding a putative 494-amino acid protein, a 5’ untranslated region of 47 bp, and a 3’ untranslated region of 545 bp (Figure S1D). The *Lvcaspase4* cDNA was 1,634 bp and contained a 1,176-bp ORF encoding a putative 496-amino acid protein, a 5’ untranslated region of 59 bp, and a 3’untranslated region of 399 bp (Figure S1B). The *Lvcaspase5* cDNA was 1,161 bp and contained an 873-bp ORF encoding a putative 290-amino acid protein, a 5’ untranslated region of 246 bp, and a 3’ untranslated region of 42 bp (Figure S1C). Based on the sequence identities and domain structures, we identified Lvcaspase2 and Lvcaspase5 as effector caspases, while Lvcaspase3 and Lvcaspase4 were initiator caspases (Figure S2). 

### 3.2: Phylogenetic tree construction

Phylogenetic analysis of caspase family proteins showed that Lvcaspase1 (*Penaeus vannamei* cas-3), Pmcaspase1 (PmCasp) and Fmcaspase1 clustered in a group (Figure S3). Lvcaspase2, Pmcaspase2 (Pm caspase) and Lvcaspase5 clustered in another group; Lvcaspase3, Mjcaspase3 (PjCaspase) and DmNedd2 clustered in a third group; and Lvcaspase4 and Dmdream clustered in a group (Figure S3). These results also revealed that Lvcaspase4 is a completely novel type of shrimp caspase. 

### 3.3: Tissue distribution of *Lvcaspase2-5*

**Figure 1:**
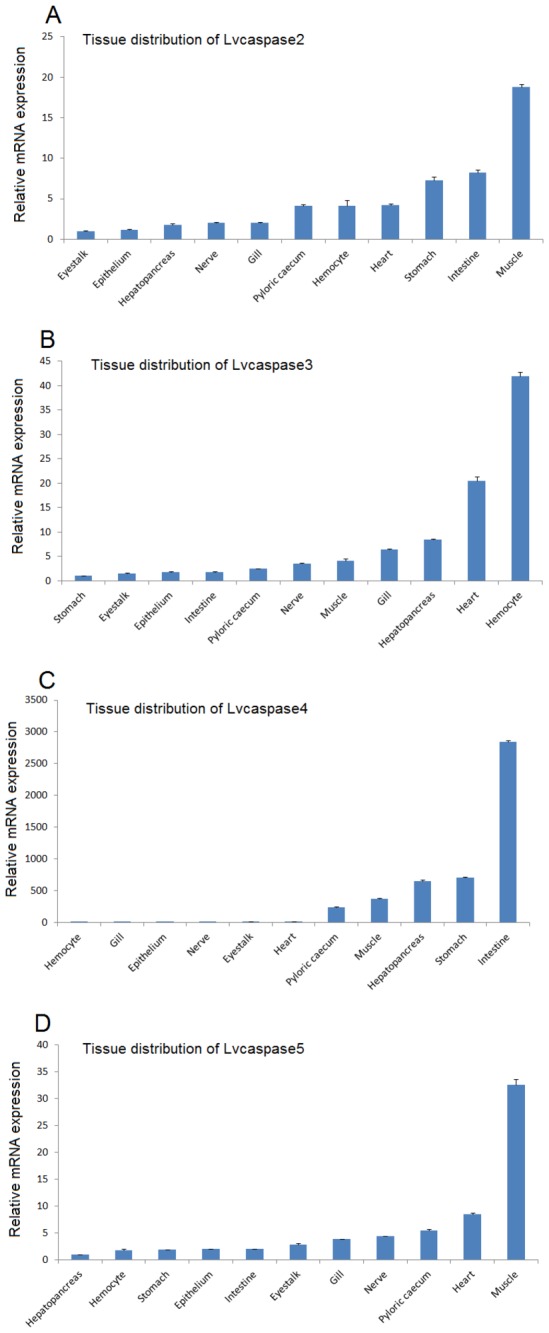
Tissue distribution of *Lvcaspase2* (A), *Lvcaspase3* (B), *Lvcaspase4* (C) and *Lvcaspase5* (D) in healthy shrimp. Hemocyte, eyestalk, gill, heart, hepatopancreas, stomach, intestine, nerve, muscle, pyloric cecum, and epithelium samples were collected from healthy *L. vannamei* for tissue distribution analysis. Shrimp total RNA was isolated and cDNA PCR templates were prepared as previously described [29-32], and 1 µl of a 5-fold dilution of cDNA template was used to determine the expression levels of *Lvcaspase2-5* in various tissues using qPCR. The expression of *Lvcaspase2* in eyestalk, the expression of *Lvcaspase3* in hepatopancreas, the expression of *Lvcaspase4* in hemocyte and the expression of *Lvcaspase5* in stomach were set as 1.0. qPCR was performed on three replicates per sample. Data are expressed as the means ± S.E. (n =3).

In healthy shrimp, when normalized to the mRNA expression level in the eyestalk (1.00-fold), *Lvcaspase2* was expressed at a higher level in epithelium (1.15-fold), hepatopancreas (1.81-fold increase), nerve (2.03-fold), gill (2.06-fold), pyloric cecum (4.09-fold), heart (4.17-fold), hemocytes (4.17-fold), stomach (7.24-fold), intestine (8.21-fold), and muscle (18.81-fold) (Figure 1A); *Lvcaspase3* was highly expressed in eyestalk (1.53-fold), epithelium (1.79-fold), intestine (1.86-fold), pyloric cecum (2.48-fold), nerve (3.51-fold), muscle (4.18-fold), gill (6.39-fold), hepatopancreas (8.40-fold), heart (20.53-fold), and hemocytes (41.92-fold) when normalized to the mRNA expression level in the stomach (1.00-fold) (Figure 1B); *Lvcaspase4* was highly expressed in gill (3.43-fold), epithelium (4.41-fold), nerve (8.82-fold), eyestalk (9.94-fold), heart (14.86-fold), pyloric cecum (237.18-fold), muscle (366.40-fold), hepatopancreas (654.03-fold increase), stomach (706.68-fold), and intestine (2843.09-fold) when normalized to the mRNA expression level in hemocytes (1.00-fold) (Figure 1C); and *Lvcaspase5* was highly expressed in hemocytes (1.88-fold), stomach (1.90-fold), epithelium (2.03-fold), intestine (2.06-fold), eyestalk (2.92-fold), gill (3.90-fold), nerve (4.41-fold), pyloric cecum (5.55-fold), heart (8.51-fold), and muscle (32.51-fold) when normalized to the mRNA expression level in the hepatopancreas (1.00-fold) (Figure 1D).

### 3.4: Expression profiles of *Lvcaspase2-5* after WSSV challenges

**Figure 2:**
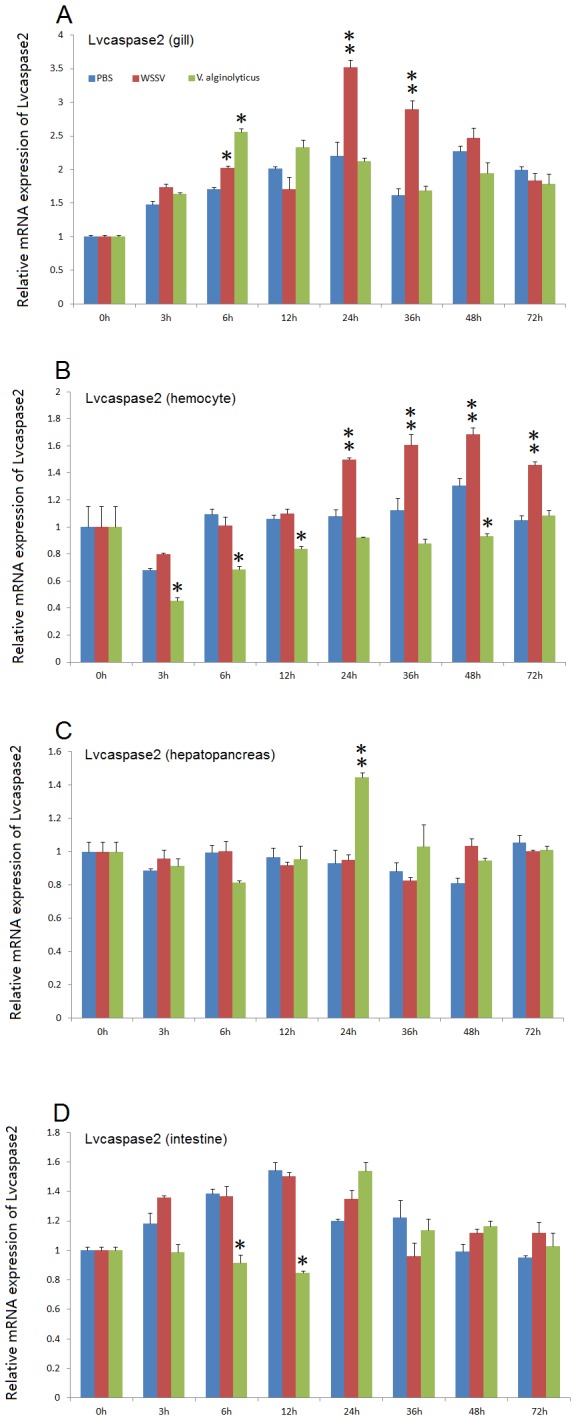
Temporal expression patterns of *Lvcaspase2* in the gill (A), hepatopancreas (B), hemocyte (C) and intestine (D) after PBS, WSSV and *V. alginolyticus* injection. Healthy *L. vannamei* were injected intramuscularly at the third abdominal segment with PBS, *V. alginolyticus* or WSSV inocula. Gill, hemocyte, hepatopancreas, and intestine samples were collected at the indicated time points. The expression levels of *Lvcaspase2* in the tissues of immune-challenged shrimp were determined by qPCR analysis. The expression of *Lvcaspase2* in the untreated shrimp (0 hpi) was set as 1.0. The mRNA expression levels of *Lvcaspase2* were normalized to those of *LvEF-1α* using the relative standard curve method. qPCR was performed on three replicates per sample. Data are expressed as the means ± S.E. (n =3).

**Figure 3:**
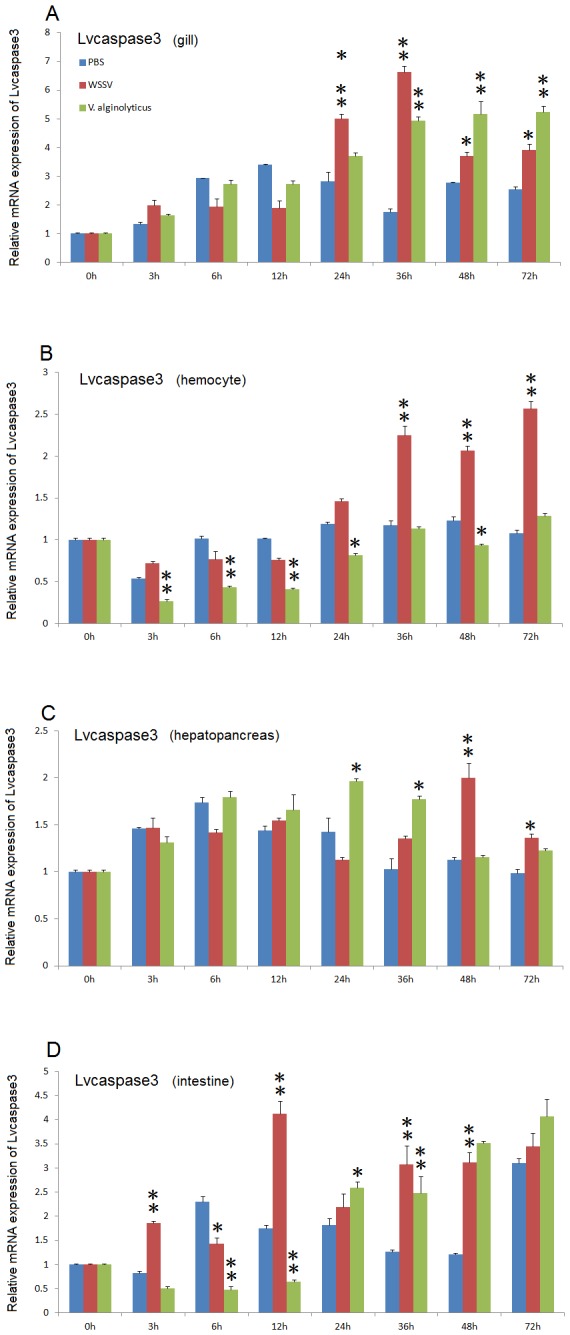
Temporal expression patterns of *Lvcaspase3* in the gill (A), hepatopancreas (B), hemocyte (C) and intestine (D) after PBS, WSSV and *V. alginolyticus* injection. Healthy *L. vannamei* were injected intramuscularly at the third abdominal segment with PBS, *V. alginolyticus* or WSSV inocula. Gill, hemocyte, hepatopancreas, and intestine samples were collected at the indicated time points. The expression levels of *Lvcaspase3* in the tissues of immune-challenged shrimp were determined by qPCR analysis. The expression of *Lvcaspase3* in the untreated shrimp (0 hpi) was set as 1.0. The mRNA expression levels of *Lvcaspase3* were normalized to those of *LvEF-1α* using the relative standard curve method. qPCR was performed on three replicates per sample. Data are expressed as the means ± S.E. (n =3).

**Figure 4:**
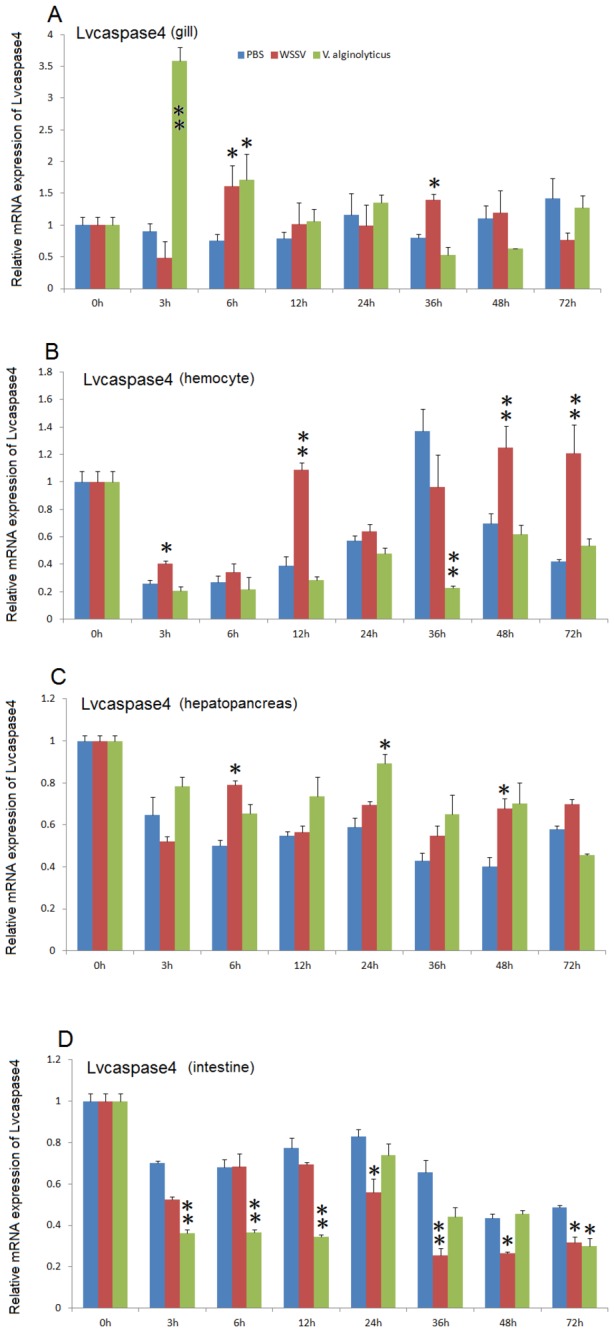
Temporal expression patterns of *Lvcaspase4* in the gill (A), hepatopancreas (B), hemocyte (C) and intestine (D) after PBS, WSSV and *V. alginolyticus* injection. Healthy *L. vannamei* were injected intramuscularly at the third abdominal segment with PBS, *V. alginolyticus* or WSSV inocula. Gill, hemocyte, hepatopancreas, and intestine samples were collected at the indicated time points. The expression levels of *Lvcaspase4* in the tissues of immune-challenged shrimp were determined by qPCR analysis. The expression of *Lvcaspase4* in the untreated shrimp (0 hpi) was set as 1.0. The mRNA expression levels of *Lvcaspase4* were normalized to those of *LvEF-1α* using the relative standard curve method. qPCR was performed on three replicates per sample. Data are expressed as the means ± S.E. (n =3).

**Figure 5:**
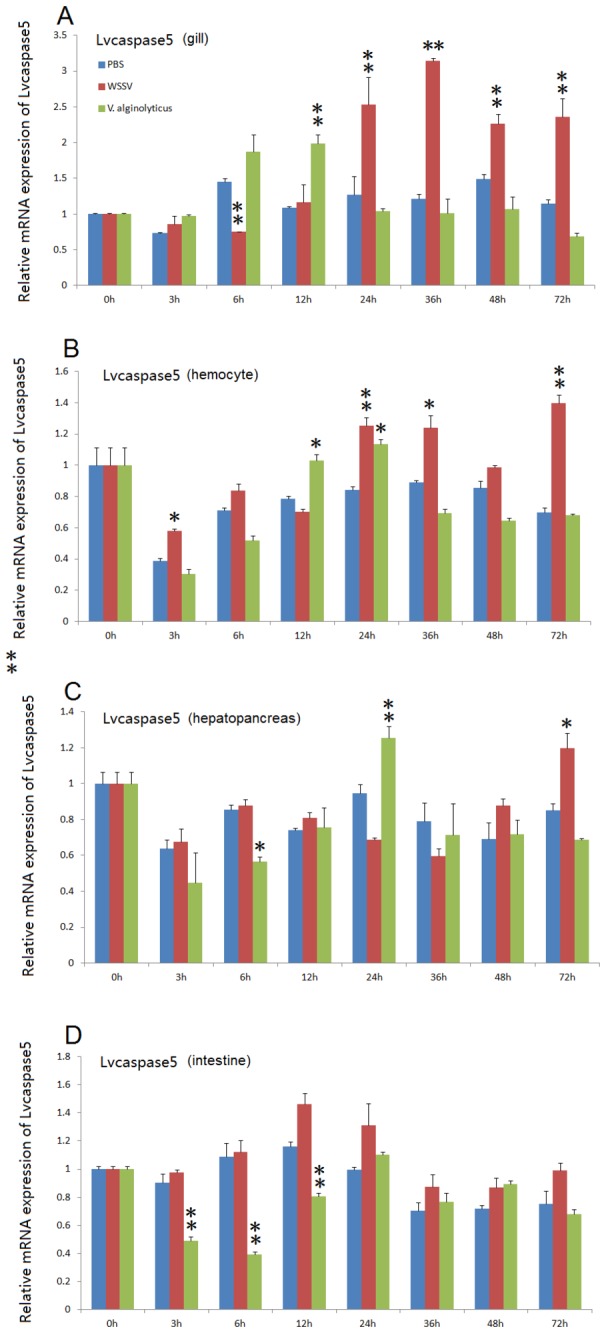
Temporal expression patterns of *Lvcaspase5* in the gill (A), hepatopancreas (B), hemocyte (C) and intestine (D) after PBS, WSSV and *V. alginolyticus* injection. Healthy *L. vannamei* were injected intramuscularly at the third abdominal segment with PBS, *V. alginolyticus* or WSSV inocula. Gill, hemocyte, hepatopancreas, and intestine samples were collected at the indicated time points. The expression levels of *Lvcaspase5* in the tissues of immune-challenged shrimp were determined by qPCR analysis. The expression of *Lvcaspase5* in the untreated shrimp (0 hpi) was set as 1.0. The mRNA expression levels of *Lvcaspase5* were normalized to those of *LvEF-1α* using the relative standard curve method. qPCR was performed on three replicates per sample. Data are expressed as the means ± S.E. (n =3).

**Figure 6:**
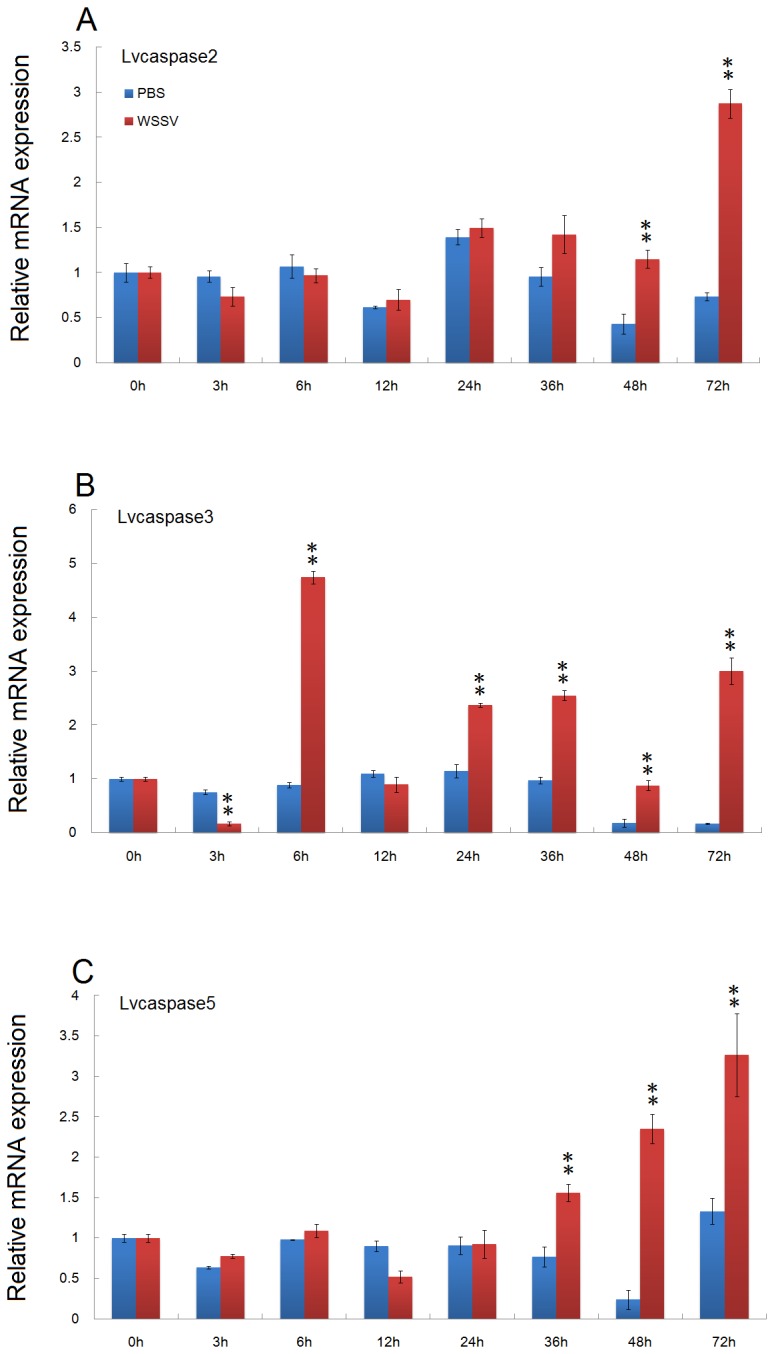
Temporal expression patterns of *Lvcaspase2* (A), *Lvcaspase3* (B) and *Lvcaspase5* (C) in the muscle after PBS and WSSV injection. Healthy *L. vannamei* were injected intramuscularly at the third abdominal segment with 100 µL of PBS (control group) or 100 µL of WSSV inoculum (10^7^ copies). At 0, 3, 6, 12, 24, 36, 48, and 72 hours post-injection (hpi), five shrimp from each group were randomly selected to take muscle samples for qPCR analysis. The expression levels in untreated shrimp (0 hpi) were set as 1.0. The mRNA expression levels of *Lvcaspase2*, *Lvcaspase3*, and *Lvcaspase5* were normalized to those of *LvEF-1α* using the relative standard curve method. qPCR was performed on three replicates per sample. Data are expressed as the means ± S.E. (n =3).

After WSSV infection, *Lvcaspase2* expression in the gill and hemocytes were increased compared with the PBS injection group, but no significant changes in *Lvcaspase2* transcript level occurred in the hepatopancreas or intestine (Figure 2). *Lvcaspase3* was upregulated in the gill, hemocytes, hepatopancreas and intestine after WSSV infection (Figure 3). *Lvcaspase4* was upregulated in the hemocytes but downregulated in the intestine after WSSV infection (Figure 4). *Lvcaspase5* was upregulated in the gill and hemocytes after WSSV infection (Figure 5). In the muscle, *Lvcaspase2*, *Lvcaspase3*, and *Lvcaspase5* were all upregulated after WSSV infection (Figure 6).

### 3.5: Subcellular localization of Lvcaspase2-5 in *Drosophila* S2 cells

**Figure 7:**
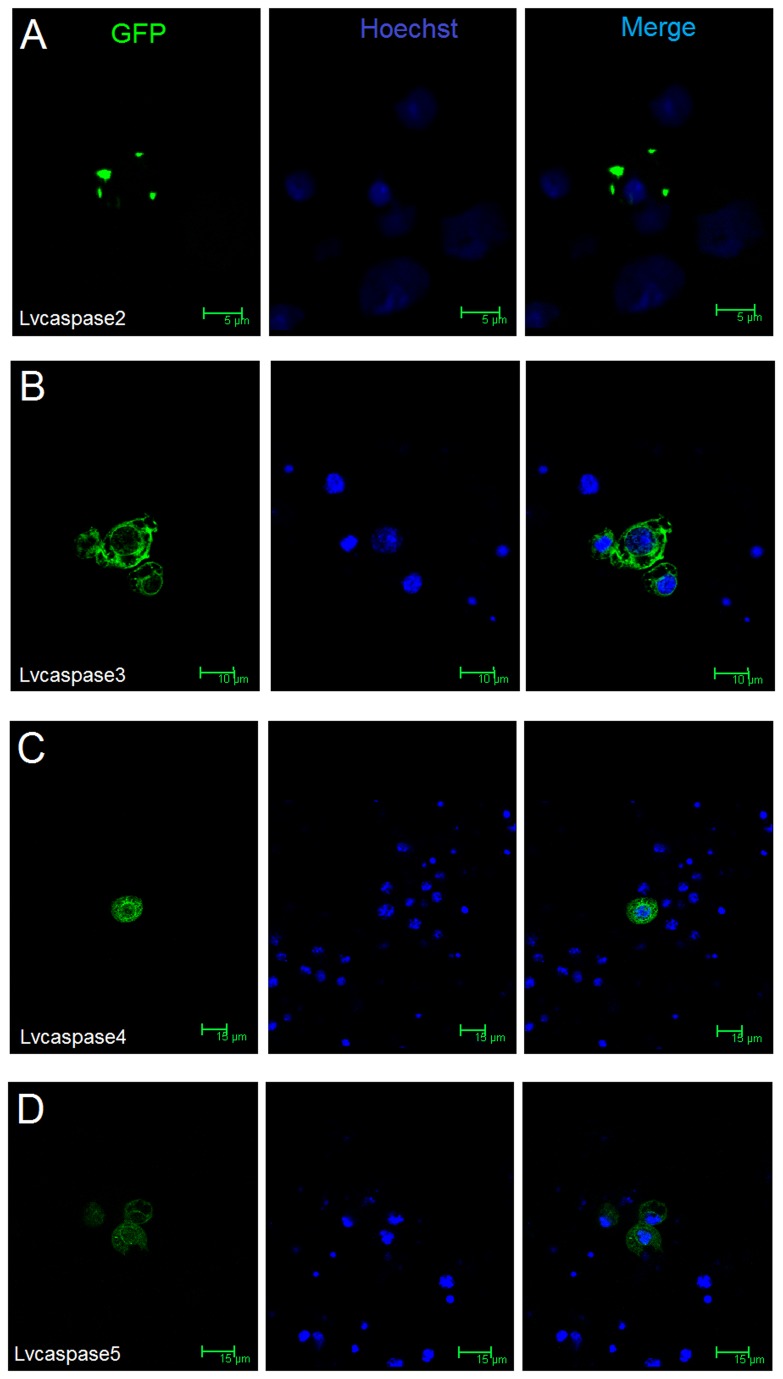
Determination of the subcellular localization patterns of Lvcaspase2 (A), Lvcaspase3 (B), Lvcaspase4 (C) and Lvcaspase5 (D) using confocal microscopy. *Drosophila* S2 cells were transfected with pAC5.1-Lvcaspase2-5-GFP. At 36 hours post-transfection, cells on the cover slips were washed twice with PBS, fixed using Immunol Staining Fix Solution (Beyotime, China) and stained with Hoechst 33258 Solution (Beyotime, China). The cells were observed using a Leica laser scanning confocal microscope as previously described [30,31,36]. .

The subcellular localization of Lvcaspase2-5 proteins may provide clues about their functions or positions in the caspase cascades. Fluorescent imaging of Lvcaspase2-GFP in *Drosophila* S2 cells showed that Lvcaspase2 was localized to the cytoplasm as speck-like aggregates near the membrane, while Lvcaspase3-5-GFP proteins localized in distinct patterns to the nucleus and cytoplasm of *Drosophila* S2 cells (Figure 7).

### 3.6: *In vivo* knock-down of *Lvcaspase2-5* by dsRNA-mediated gene silencing

**Figure 8:**
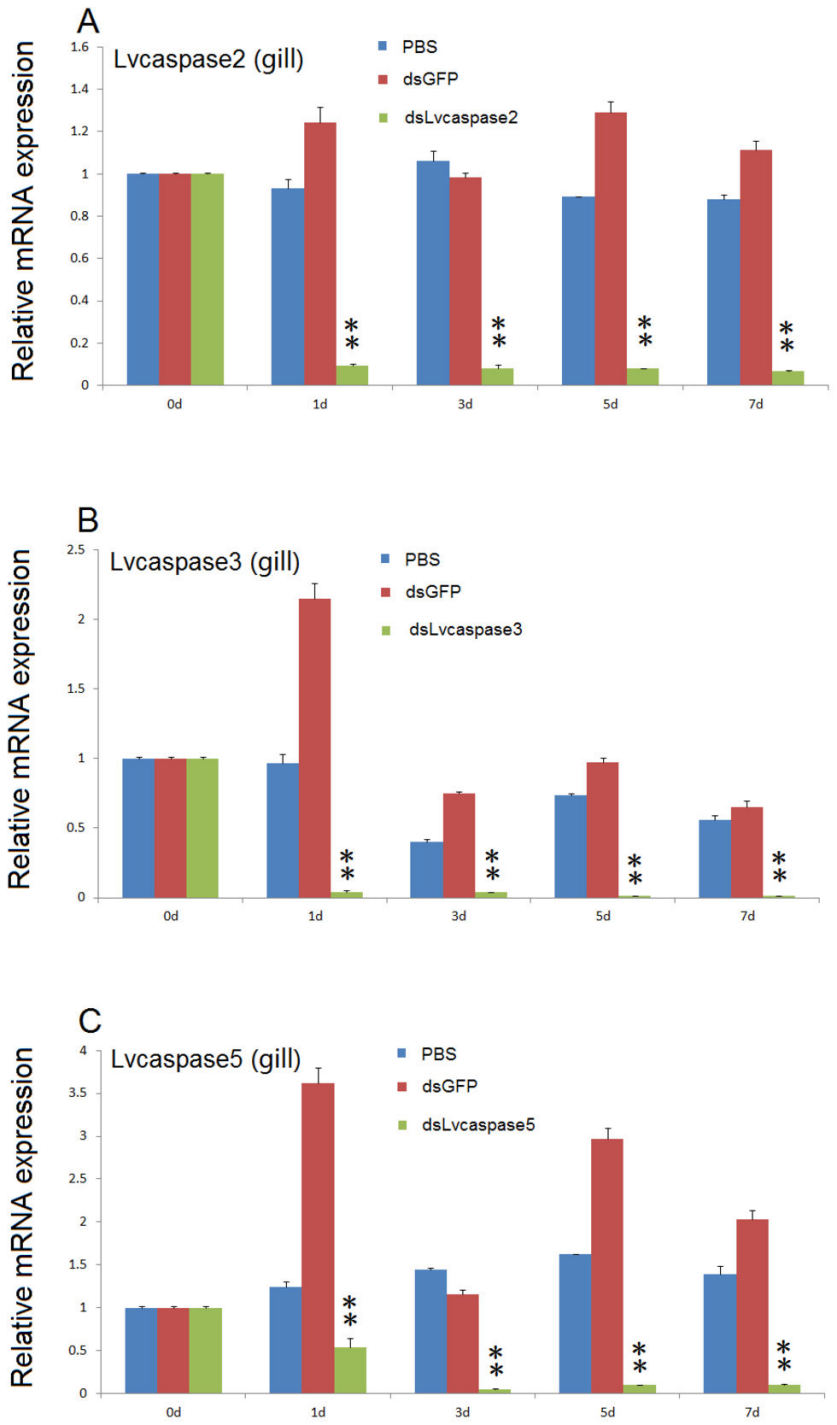
Expression of *Lvcaspase2* (A), *Lvcaspase3* (B) and *Lvcaspase5* (C) in the shrimp gill is significantly suppressed by dsRNA-mediated RNAi. At the indicated time points after PBS, dsGFP (control), dsLvcaspase2, dsLvcaspase3 or dsLvcaspase5 injection, total RNA was extracted from the gill and reverse transcribed to cDNA. The expression levels of *Lvcaspase2* (A)*, Lvcaspase3* (B) and *Lvcaspase5* (C) were determined using qPCR. Their expression levels in untreated shrimp (0 hpi) were set as 1.0. qPCR was performed in triplicate for each sample. Statistical significance was evaluated using Student’s *t*-test (*, p < 0.05; **, p < 0.01).

Using dsRNA-mediated gene silencing, we successfully suppressed the expression of *Lvcaspase2, Lvcaspase3 and Lvcaspase5*, but not *Lvcaspase4*, in the gill, as previously described [37]. Using qPCR, we observed that *Lvcaspase2, Lvcaspase3* and *Lvcaspase5* transcripts in the gill were significantly reduced at 24, 72, 120 and 144 hpi compared with the dsGFP control group (Figure 8). 

### 3.7: Knock-down of *Lvcaspase2*, *Lvcaspase3*, and *Lvcaspase5* increases WSSV replication

**Figure 9:**
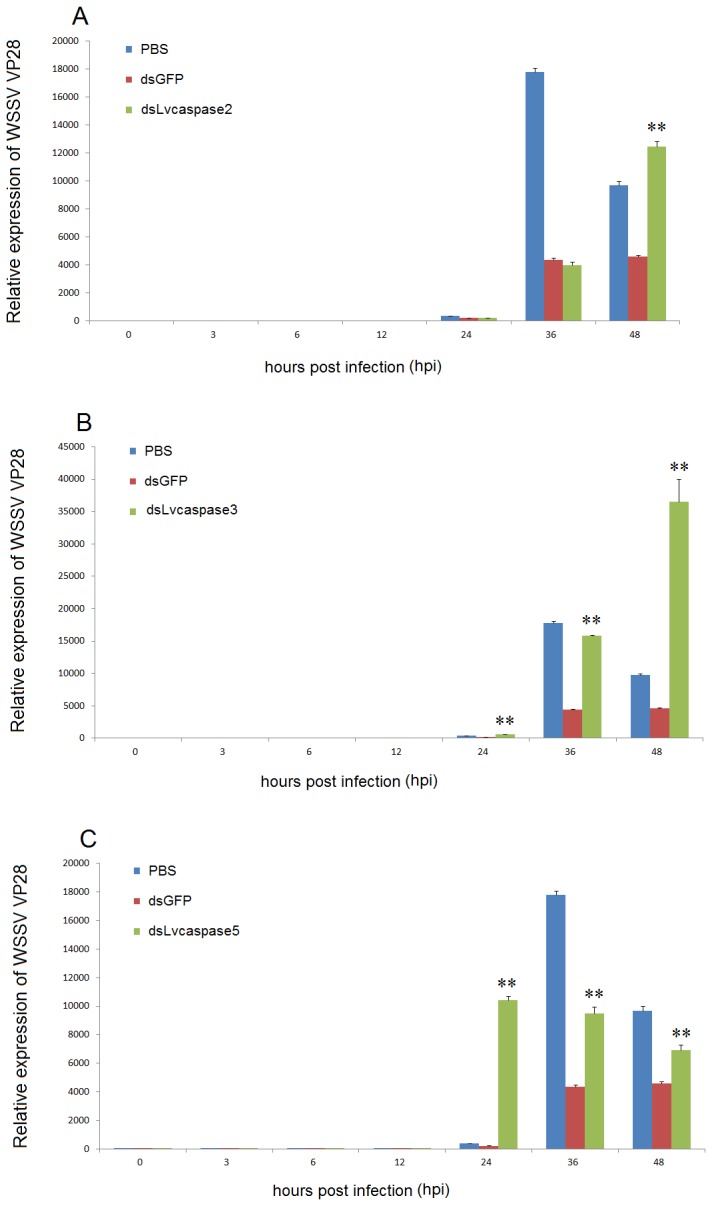
Silencing of *Lvcaspase2* (A), *Lvcaspase3* (B) or *Lvcaspase5* (C) facilitates replication of WSSV. *L. vannamei* were intramuscularly injected with 100 µl WSSV inoculum (approximately 10^7^ copies/shrimp) 48 hours after dsRNA injection, and gill samples were collected at the indicated time points. The expression levels of WSSV VP28 in gills from WSSV-infected shrimp injected 48 hours earlier with PBS, dsGFP (control), dsLvcaspase2, dsLvcaspase3 or dsLvcaspase5 were determined using qPCR. qPCR was performed in triplicate for each sample. Statistical significance was evaluated using Student’s *t*-test (*, p < 0.05; **, p < 0.01).

To further evaluate the role of Lvcaspase2, Lvcaspase3 and Lvcaspase5 in the shrimp defense against WSSV infection, we performed WSSV infection experiments in dsRNA-injected *L. vannamei*. When *L. vannamei* were infected with WSSV 48 hours after dsRNA injection, we found that at 48 hours post-infection (hpi), but not at 24 or 36 hpi, the expression of WSSV *VP28* in the gill from the dsLvcaspase2-injection group was dramatically higher than in the dsGFP- and PBS- injection groups (Figure 9A). At 36 and 48 hpi (but not 24 hpi), the expression of WSSV *VP28* in the gill from the dsLvcaspase3-injection group was dramatically higher than in the dsGFP- and PBS-injection groups (Figure 9B). At 24, 36 and 48 hpi, the expression of WSSV *VP28* in the gill from the dsLvcaspase5-injection group was dramatically higher than in the dsGFP- and PBS-injection groups (Figure 9C). We also noticed that at 24 hpi, the expression of *VP28* was very low in the PBS-, dsGFP-, dsLvcaspase2- and dsLvcaspase3-injection groups but was very high in the dsLvcaspase5-injection group (Figure 9). This result suggests that silencing *Lvcaspase5* might accelerate WSSV infection. Collectively, these data suggest that Lvcaspase2, Lvcaspase3 and Lvcaspase5 are all involved in the host defense against WSSV infection but have different roles. 

## Discussion

There are two distinct apoptotic pathways in mammals: the extrinsic pathway (or death receptor pathway) and the intrinsic pathway (or mitochondria/cytochrome c pathway) [6,38,39]. In the extrinsic pathway, binding of the death ligand to a death receptor such as TNFα-TNFR leads to death receptor-FADD-procaspase-8 complex formation, thereby resulting in the cleavage and activation of caspase-8 [6,11,40]. The downstream effector caspase-3 is then activated, ultimately resulting in cell death [6,7,11,40]. Intracellular signals such as DNA damage, oxidative stress and viral infection can activate the intrinsic pathway [6,11,40]. All these signals converge on the mitochondria, which then release cytochrome c into the cytoplasm [6,11,39]. The cytochrome c binds to Apaf-1 and forms the apoptosome, which can interact with and activate procaspase-9 [6,7,11,39]. The activated caspase-9 initiates the caspase cascade, allowing the downstream effector caspases to execute the destruction of the cell [6,7,11,39]. The extrinsic and intrinsic pathways converge at the point of activating the effector caspases [6,11,39]. Thus, caspases are central regulators of apoptosis. 

In *Caenorhabditis elegans*, however, the mammalian extrinsic pathway seems not to exist, as this species lacks essential components of this pathway [11]. Although *Drosophila* encodes homologs of a mammalian death ligand and receptor (called Eiger and Wengen in *Drosophila*, respectively), the receptor Wengen lacks the death domain to transduce death signaling, suggesting that *Drosophila* may not have functional extrinsic apoptosis pathway [11,41,42]. In invertebrates, which lack adaptive immunity, programmed cell death (i.e., apoptosis) functions as an important immune response against pathogen infection [43]. In our previous studies, we cloned the TNF superfamily (*LvTNFSF*) gene and the TNFR superfamily (*LvTNFRSF*) gene from *L. vannamei*, and we found that LvTNFRSF, like *Drosophila* Wengen, lacks the death domain to transduce death signaling [30]. Therefore, shrimp may rely mainly on the intrinsic pathway for apoptosis-mediated immune responses. 

Five caspase genes have been reported in penaeid shrimp that are extremely sensitive to WSSV: *Penaeus merguiensis* cap-*3*, *Penaeus vannamei cas-3* (called *Lvcaspase1* in this study), *PjCaspase* from *P. japonicas* (called *Mjcaspase3* in this study), *PmCasp* and *Pm caspase from P. monodon* (called *Pmcaspase1 and Pmcaspase2*, respectively, in this study) [11]. These five shrimp caspases fall into 3 different types: caspase-1 type, caspase-2 type, and caspase-3 type (Figure S3). To further investigate function of the caspase family proteins in the host defense against WSSV infection, we cloned four novel caspases from *L. vannamei* in this study. Lvcaspase2-5 show the typical domain structure of caspase family proteins, with the conserved consensus motifs p20 and p10 (Figure S2). Like *Pmcaspase1* (*PmCasp*) and *Mjcaspase3* (*PjCaspase*), expression of *Lvcaspase2-5* mRNA can be induced by WSSV infection but show distinct patterns. *Lvcaspase2* mRNA is induced in the gill and in hemocytes (Figure 2); *Lvcaspase3* mRNA is induced in all the tissues detected in our study including the gill, hepatopancreas, hemocytes, intestine and muscle (3 and 9); *Lvcaspase4* mRNA was mainly induced in the hepatopancreas and hemocytes (Figure 4); and *Lvcaspase5* mRNA was induced in the gill, hepatopancreas, hemocytes and muscle (Figures 5 and 9). The different expression patterns observed after WSSV infection may suggest that Lvcaspase2-5 play different roles in host defense. 

Although shrimp caspases have the signature p20 and p10 domains of the caspase family proteins, their sequence identities with mammalian caspases are not high enough for sequence-based classification into existing caspase classes. In this study, we named shrimp caspases based on their reported orders. According to our analysis, Lvcaspase1 (*Penaeus vannamei* cas-3), Pmcaspase1 (PmCasp) and Pmcaspase2 (Pm caspase) are effector caspases, and Mjcaspase3 (PjCaspase) is an initiator caspase (Figure S2). The domain structures of Lvcaspase2-5 indicated that Lvcaspase2 and Lvcaspase5 are effector caspases, while Lvcaspase3 and Lvcaspase4 are initiator caspases (Figure S2). 

Although five shrimp caspases have been reported, until now their cellular localization has remained unknown. Using confocal microscopy, we found that Lvcaspase2-GFP appeared as speck-like aggregates in the cytoplasm near the membranes of *Drosophila* S2 cells, while Lvcaspase3-5-GFP localized with distinct patterns to the nucleus and cytoplasm of *Drosophila S2* cells (Figure 7). The different cellular localization patterns of GFP-tagged Lvcaspase2-5 may suggest different roles or positions in the caspase cascade. 

Silencing *Mjcaspase3* (*PjCaspase*) resulted in increased WSSV virus copy number, indicating a requirement of *Mjcaspase3* in apoptotic responses against viral infection [22]. Recently, the same group also found that the sequence diversification of *Mjcaspase3* could generate a specifically antiviral defense against WSSV infection [43]. Pmcaspase2 (Pm caspase) from *P. monodon* can induce apoptosis in SF9 insect cells, and the apoptotic activity can be blocked by AAP-1 (ORF390 or WSSV449) [19]. Further studies confirmed that AAP-1 (ORF390 or WSSV449) can directly bind to and be cleaved by Pmcaspase2 (Pm caspase), thereby inhibiting Pmcaspase2 (Pm caspase) activity [18]. Lvcaspase2 shows high similarity to Pmcaspase2 (Figure S3). To further investigate its function in WSSV infection, we suppressed the expression of *Lvcaspase2* using dsRNA-mediated gene silencing. We found that at 24 and 36 hpi, the expression of WSSV *VP28* in dsLvcaspase2-injected shrimp showed no obvious difference from the dsGFP-injected shrimp, but at 48 hpi, WSSV *VP28* expression in the dsLvcaspase2-injected shrimp was dramatically higher than in the dsGFP- and PBS- injected shrimp (Figure 9A). These results suggested that *Lvcaspase2* may be required for defending against WSSV infection. In future studies, we will investigate the effect of WSSV449 on the activities of these four novel caspases and will test whether they interact with each other. Lvcaspase3 is a homolog of Mjcaspase3 (PjCaspase). When *Lvcaspase3* was silenced, *VP28* expression was significantly higher than in the dsGFP control group at 36 and 48 hpi, in accord with results from Mjcaspase3 (Figure 9B) [22]. Lvcaspase4-5 are novel types of shrimp caspases. Unfortunately, we were unable to knock down the expression of *Lvcaspase4* using dsRNA-mediated gene silencing. When we suppressed the expression of *Lvcaspase5*, *VP28* expression was dramatically higher than in the dsGFP control group at 24, 36 and 48 hpi (Figure 9C). We also noticed that *Lvcaspase5* was the only caspase when knocked down could cause higher expression of *VP28* at the early infection stage of 24 hpi, suggesting a different role or position in the caspase cascade from *Lvcaspase2-3* (Figure 9). Pmcaspase2 (Pm caspase) has been targeted by small-molecule drugs to improve the apoptotic activity of shrimp hemocytes and thereby inhibit WSSV infection [11,44]. In future studies, we will investigate the detailed functions of Lvcaspase2-5 at different stages of WSSV infection. Development of drugs targeting caspases and manipulating shrimp apoptosis may provide novel strategies for the prevention and control of WSSV infections.

## Supporting Information

Figure S1 **Nucleotide and deduced amino acid sequences of Lvcaspase2 (**A**), Lvcaspase3 (**D**), Lvcaspase4 (**B**) and Lvcaspase5 (**C**) from *L. vannamei*.** The nucleotide (upper row) and deduced amino acid (lower row) sequences of Lvcaspase2-5 are shown. The initiation codon (ATG) and stop codon (TAA, TGA or TAG) are shown in bold. The caspase family p20 and p10 domains in Lvcaspase2-5 are shaded.
(TIF)

Figure S2 **Domain architectures of shrimp caspases.**The full-length protein sequences of shrimp caspases were subjected to the simple modular architecture research tool (SMART; http://smart.embl-heidelberg.de) to generate domain structures. The p20 and p10 domain are indicated as elliptical boxes, and the prodomain upstream of the p20 domain is indicated as a line. The initiator caspases have a long prodomain (> 90 amino acids) containing specific protein-protein interaction motifs that are necessary for their activation, whereas the effector caspases usually have a short prodomain of only 20-30 residues [8].
(TIF)

Figure S3 **A phylogenetic tree of Lvcaspase2-5 with other caspase family proteins.**The full-length amino acid sequences of caspase family proteins from typical organisms were aligned using the ClustalX2.0 program (http://www.ebi.ac.uk/tools/clustalw2). The rooted tree was then constructed by the “neighbor-joining” method and was bootstrapped 1,000 times using MEGA 4.0 software (http://www.megasoftware.net/index.html). The numbers at the nodes indicate bootstrap values. Lvcaspase2-5 are boxed in blue lines. Lvcasp1, *L. vannamei* caspase1 (Accession no. ABK88280); Lvcasp2, *L. vannamei* caspase2 (Accession no. KC660102); Lvcasp3, *L. vannamei* caspase3 (Accession no. KC660103); Lvcasp4, *L. vannamei* caspase4 (Accession no. KC660105); Lvcasp5, *L. vannamei* caspase5 (Accession no. KC660104); Pmcasp1, *Penaeus monodon* caspase1 (Accession no. AEW91437); Mjcasp3, *Marsupenaeus japonicus* caspase3 (Accession no. ABK62771); Pmcasp2, Penaeus monodon caspase2 (Accession no. ABO38430); Hscasp1, *Homo sapiens* caspase1 (Accession no. **NP_001214**); Mmcasp1, *Mus musculus* caspase1 (Accession no. **NP_033937**); Hscasp2, *H. sapiens* caspase2 (Accession no. AAH02427); Mmcasp2, *M. musculus* caspase2 (Accession no. **NP_031636**); Hscasp3, *H. sapiens* caspase3 (Accession no. **NP_116786**); Mmcasp3, *M. musculus* caspase3 (Accession no. **NP_033940**); Hscasp4, *H. sapiens* caspase4 (Accession no. **NP_001216**); Hscasp5, *H. sapiens* caspase5 (Accession no. **NP_001129584**); Hscasp6, *H. sapiens* caspase6 (Accession no. **NP_001217**); Hscasp7, *H. sapiens* caspase7 (Accession no. **NP_001253987**); Mmcasp7, *M. musculus* caspase7 (Accession no. **NP_031637**); Hscasp8, *H. sapiens* caspase8 (Accession no. **NP_001073594**); Hscas9, *H. sapiens* caspase9 (Accession no. **NP_127463**); Hscasp10, *H. sapiens* caspase10 (Accession no. AAD28403); Hscasp14, *H. sapiens* caspase14 (Accession no. **NP_036246**); DmIce, *Drosophila melanogaster* Ice (Accession no. **NP_524551**); Dmcasp1, *D. melanogaster* caspase1 (Accession no. AAB58237); Dmdream, *D. melanogaster* dream (Accession no. **NP_610193**); Dmdeath, *D. melanogaster* death executioner caspase (Accession no. **NP_477462**); DmNedd2, *D. melanogaster* Nedd2 (Accession no. **NP_524017**); Drcasp1, *D. rerio* caspase1 (Accession no. **NP_571580**); Drcasp2, *D. rerio* caspase2 (Accession no. **NP_001036160**); Drcasp3, *D. rerio* caspase3 (Accession no. **NP_571952**); Drcasp6, *Danio rerio* caspase6 (Accession no. **NP_001018333**); Drcasp7, *D. rerio* caspase7 (Accession no. **NP_001018443**); Drcasp7-2, *D. rerio* caspase7 like (Accession no. **XP_002667104**); Drcasp8, *D. rerio* caspase8 (Accession no. **NP_571585**); Drcasp9, *D. rerio* caspase9 (Accession no. **NP_001007405**); Ggcasp1, *Gallus gallus* caspase1 (Accession no. **XP_003642432**); Ggcasp2, *G. gallus* caspase2 (Accession no. **NP_001161173**); Ggcasp3, *G. gallus* caspase3 (Accession no. **NP_990056**); Ggcasp6, *G. gallus* caspase6 (Accession no. **NP_990057**); Ggcasp7, *G. gallus* caspase7 (Accession no. **XP_421764**); Ggcasp8, *G. gallus* caspase8 (Accession no. **NP_989923**); Ggcasp9, *G. gallus* caspase9 (Accession no. **XP_424580**); Ggcasp10, *G. gallus* caspase10 (Accession no. **XP_421936**); Ggcasp18, *G. gallus* caspase18 (Accession no. **NP_001038154**); Xlcasp1, *X. laevis* caspase1 (Accession no. **NP_001081223**); Xlcasp2, *X. laevis* caspase2 (Accession no. **NP_001081404**); Xlcasp3, Xenopus laevis caspase3 (Accession no. **NP_001081225**); Xlcasp7, *X. laevis* caspase7 (Accession no. **NP_001081408**); Xlcasp6, *X. laevis* caspase6 (Accession no. **NP_001081406**); Xlcasp8, *X. laevis* caspase8 (Accession no. **NP_001079034**); Xlcasp9, *X. laevis* caspase9 (Accession no. **NP_001079035**); Xlcasp10, *X. laevis* caspase10 (Accession no. **NP_001081410**); CeCED3, *Caenorhabditis elegans* CED3 (Accession no. **NP_001255708**); CeCSP1, *C. elegans* CSP1 (Accession no. **NP_001022452**); CeCSP2, *C. elegans* CSP2 (Accession no. **NP_001023575**).
(TIF)
