# Supplementary figures and images for: Characterization of Four Novel Caspases from Litopenaeus vannamei (Lvcaspase2-5) and Their Role in WSSV Infection through dsRNA-Mediated Gene Silencing
Source: PLoS One. 2013 Dec 23;8(12):e80418. doi: 10.1371/journal.pone.0080418 (PMC3871164; doi:10.1371/journal.pone.0080418)

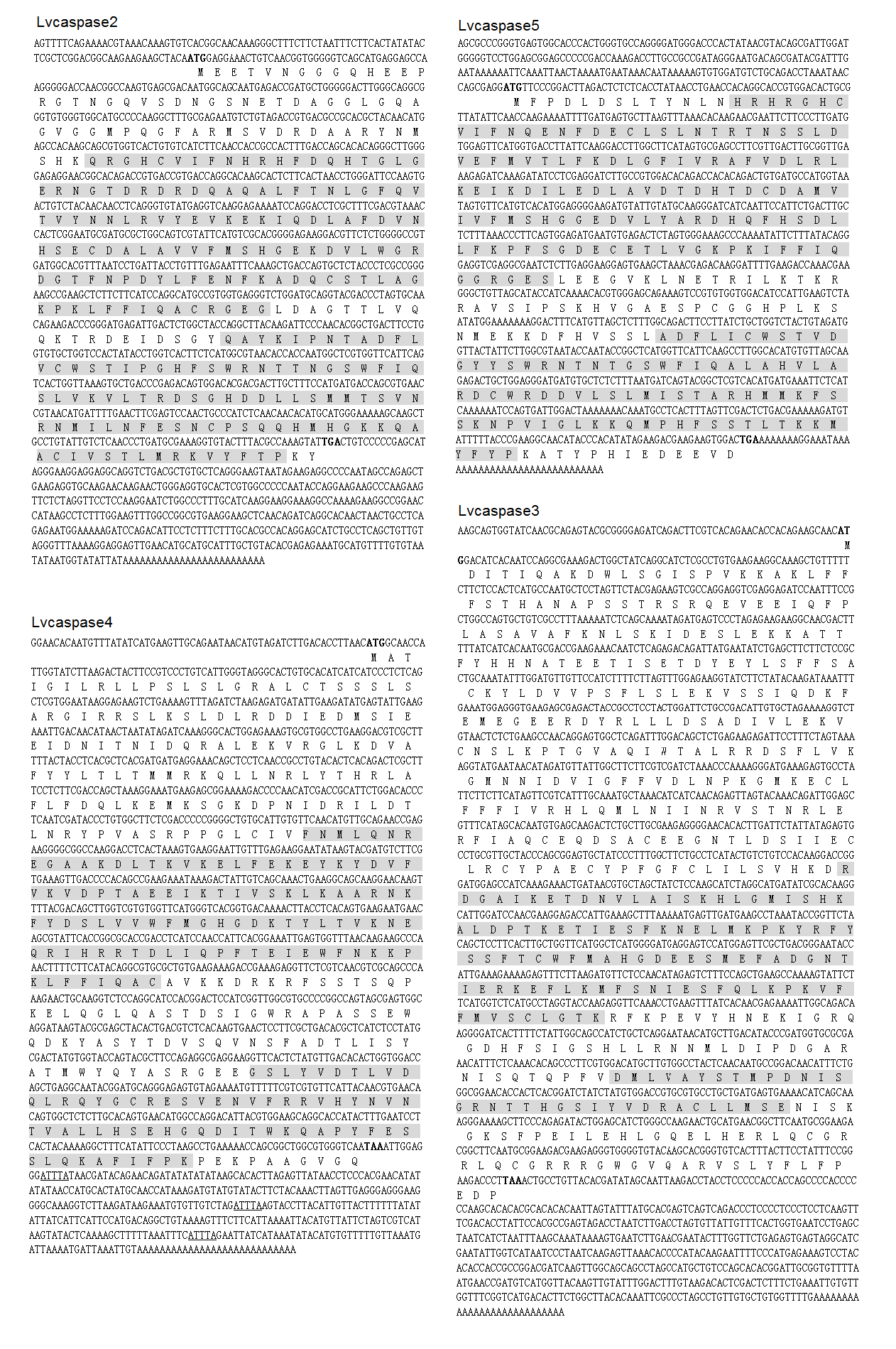

Supplement: Figure S1 — Nucleotide and deduced amino acid sequences of Lvcaspase2 (A), Lvcaspase3 (D), Lvcaspase4 (B) and Lvcaspase5 (C) from L. vannamei. The nucleotide (upper row) and deduced amino acid (lower row) sequences of Lvcaspase2-5 are shown. The initiation codon (ATG) and stop codon (TAA, TGA or TAG) are shown in bold. The caspase family p20 and p10 domains in Lvcaspase2-5 are shaded. (TIF) [file pone.0080418.s001.tif]

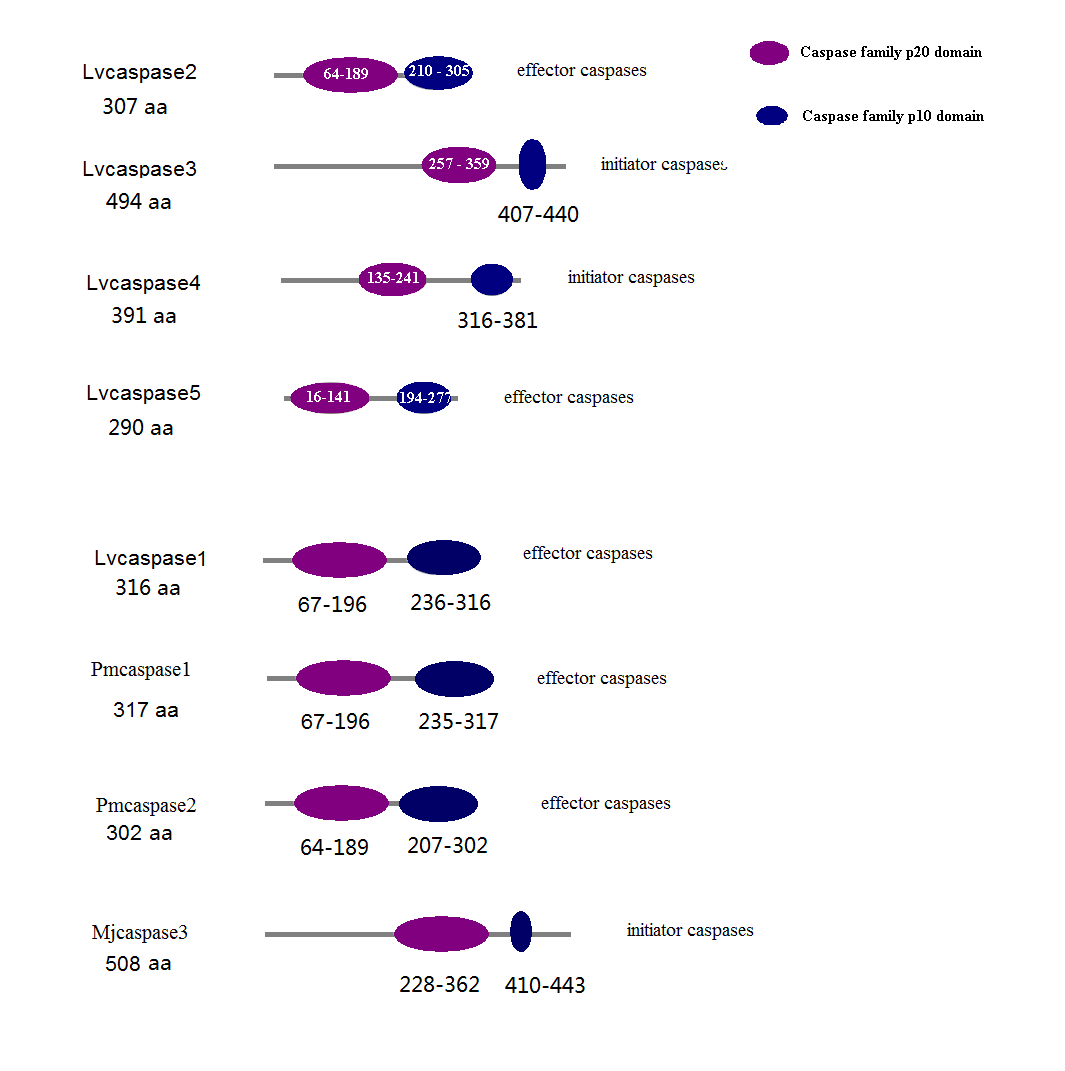

Supplement: Figure S2 — Domain architectures of shrimp caspases.The full-length protein sequences of shrimp caspases were subjected to the simple modular architecture research tool (SMART; http://smart.embl-heidelberg.de) to generate domain structures. The p20 and p10 domain are indicated as elliptical boxes, and the prodomain upstream of the p20 domain is indicated as a line. The initiator caspases have a long prodomain (> 90 amino acids) containing specific protein-protein interaction motifs that are necessary for their activation, whereas the effector caspases usually have a short prodomain of only 20-30 residues [8]. (TIF) [file pone.0080418.s002.tif]

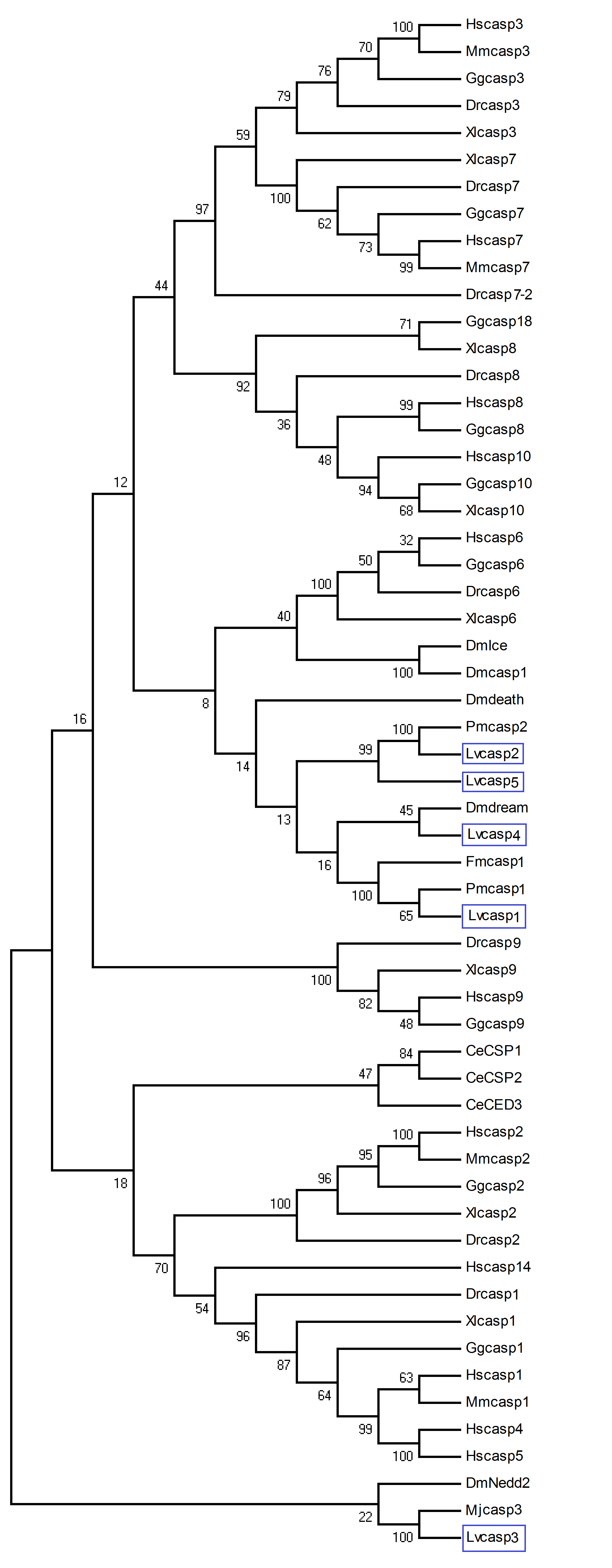

Supplement: Figure S3 — A phylogenetic tree of Lvcaspase2-5 with other caspase family proteins.The full-length amino acid sequences of caspase family proteins from typical organisms were aligned using the ClustalX2.0 program (http://www.ebi.ac.uk/tools/clustalw2). The rooted tree was then constructed by the “neighbor-joining” method and was bootstrapped 1,000 times using MEGA 4.0 software (http://www.megasoftware.net/index.html). The numbers at the nodes indicate bootstrap values. Lvcaspase2-5 are boxed in blue lines. Lvcasp1, L. vannamei caspase1 (Accession no. ABK88280); Lvcasp2, L. vannamei caspase2 (Accession no. KC660102); Lvcasp3, L. vannamei caspase3 (Accession no. KC660103); Lvcasp4, L. vannamei caspase4 (Accession no. KC660105); Lvcasp5, L. vannamei caspase5 (Accession no. KC660104); Pmcasp1, Penaeus monodon caspase1 (Accession no. AEW91437); Mjcasp3, Marsupenaeus japonicus caspase3 (Accession no. ABK62771); Pmcasp2, Penaeus monodon caspase2 (Accession no. ABO38430); Hscasp1, Homo sapiens caspase1 (Accession no. NP_001214); Mmcasp1, Mus musculus caspase1 (Accession no. NP_033937); Hscasp2, H. sapiens caspase2 (Accession no. AAH02427); Mmcasp2, M. musculus caspase2 (Accession no. NP_031636); Hscasp3, H. sapiens caspase3 (Accession no. NP_116786); Mmcasp3, M. musculus caspase3 (Accession no. NP_033940); Hscasp4, H. sapiens caspase4 (Accession no. NP_001216); Hscasp5, H. sapiens caspase5 (Accession no. NP_001129584); Hscasp6, H. sapiens caspase6 (Accession no. NP_001217); Hscasp7, H. sapiens caspase7 (Accession no. NP_001253987); Mmcasp7, M. musculus caspase7 (Accession no. NP_031637); Hscasp8, H. sapiens caspase8 (Accession no. NP_001073594); Hscas9, H. sapiens caspase9 (Accession no. NP_127463); Hscasp10, H. sapiens caspase10 (Accession no. AAD28403); Hscasp14, H. sapiens caspase14 (Accession no. NP_036246); DmIce, Drosophila melanogaster Ice (Accession no. NP_524551); Dmcasp1, D. melanogaster caspase1 (Accession no. AAB58237); Dmdream, D. melanogaster dream (Accession no. NP_61019 [file pone.0080418.s003.tif]
